# Correction: Moving towards a core measures set for patient safety in perioperative care: An e-Delphi consensus study

**DOI:** 10.1371/journal.pone.0317063

**Published:** 2025-01-02

**Authors:** J. P. Dinis-Teixeira, Ana Beatriz Nunes, Andreia Leite, Willemijn L. A. Schäfer, Claudia Valli, Ismael Martínez-Nicolas, Ayshe Seyfulayeva, Pedro Casaca Carvalho, Anna Rodríguez, Daniel Arnal-Velasco, Irene Leon, Carola Orrego, Paulo Sousa, on behalf of the SAFEST Consortium and the SAFEST Scientific Advisory Group

There are errors in the Acknowledgments section. The correct Acknowledgments section is as follows: The authors would like to commend the SAFEST consortium members for their contribution: Daniel Arnal-Velasco, Joaquim Baneres, Ashish Bartakke, Hiske Calsbeek, Genis Carrasco, Pedro Casaca-Carvalho, Edoardo De Robertis, Yvette Emond, Neus Fabregas, Javier García-Silva, Pascal Garel, Oliver Groene, Anita Heideveld-Chevalking, Mari Kangasniemi, Janne Kommusaar, Kaja Kristensen, Andreia Leite, Irene Leon, Ismael Martínez-Nicolás, David Marx, Marie Nabbe, Ana Beatriz Nunes, Carola Orrego, Kaja Polluste, Janne Pühvel, Eva Romero-Garcia, Yolanda Sanduende-Otero, Willemijn Schäfer, Caroline Schlinkert, Ayshe Seyfulayeva, Victor Soria-Aledo, Paulo Sousa, Joel Starkopf, Rosa Sunol, Helena Vall, Claudia Valli, Nina van der Schoot, Lilian Van Tuyl, Frantisek Vlcek, Marieke Voshaar, Cordula Wagner, Sophie Wang, Adam Žaludek, and Sandro Zamarian.

The authors would also like to acknowledge the SAFEST Scientific Advisory Group members for contributing to this study: Aamer Ahmed, Fragkiskos Angelis, Catarina Baptista, Metaxia Bareka, Kateryna Bielka, Mercedes Bilbao, Federico Bilotta, Elvira Bisbe, Hans D de Boer, Dialina Brilhante, Pedro Carrascal, Pedro Delgado, Zsuzsanna Farkas-Pall, Aidan Fowler, Loredana Gigli, Helen Haskell, Arvid Steinar Haugen, Jan Hofland, Beverley Hunt, Ib Jammer, Janek Kapper, Marion van der Kolk, Natasa Kovac, Susana Lorenzo, Rui Malheiro, Xose Manuel Meijome, Jannicke Mellin-Olsen, Margaret Murphy, Maria Ntalouka, Marta Dora Ornelas, Maria Papadakaki, Danica Rotar Pavlic, Julien Picard, Marek Pietruszka, Benedikt Preckel, Finn Radktke, José Manuel Rodríguez, Narimantas Evaldas Samalavicius, Pedro Vieira dos Santos, Ed Schoemaker, Kawaldip Sehmi, Henriëtte Smid-Nanninga, Joel Starkopf, John Tansley, Zuzana Tomášková, Francesco Venneri, Matthias Weigl, and Argyro Zoumprouli.
