# Supplementary material for: Moving towards a core measures set for patient safety in perioperative care: An e-Delphi consensus study
Source: PLoS One. 2024 Oct 23;19(10):e0311896. doi: 10.1371/journal.pone.0311896 (PMC11498713; doi:10.1371/journal.pone.0311896)
Supplement: S1 Appendix — (DOCX) [file pone.0311896.s001.docx]

# **S1 appendix**. Final List of Indicators.

**Preoperative Indicators**

| Structure Indicators |
| --- |
| A consultant anaesthesiologist is responsible for leading the anaesthetic preoperative assessment service. |
| There is an internal policy for preoperative preparation defined and diffused among professionals, including all the following: fasting, investigations, blood typing, thromboprophylaxis, perioperative diabetes management, and allergies. |
| There is an agreed internal policy for referral pathways to other specialties to expedite further investigations that is diffused among professionals. |
| There is a defined internal policy to ensure that abnormal results of investigations are flagged to the relevant person in a timely manner defined that is diffused among professionals. |
| Process Indicators |
| Preassessment according to local recommendations is conducted at least the day before surgery. |
| A preoperative up to date medication list is available in the clinical records. |
| A preoperative glucose monitoring is conducted in diabetic patients by a knowledgeable and trained professional based on the best available evidence. |
| Re-assess of venous thromboembolism and bleeding risk using risk assessment criteria is conducted on admission and within 24 hours of admission. |
| Patients assessed to be at risk of venous thromboembolism are offered prophylaxis in accordance with best practices. |
| The stoma site is marked if indicated. |
| Risk assessment for pressure ulcers using a standardized scale upon admission is conducted. |
| Outcome Indicators |
| Proportion of prospective surgical patients that undergo electrocardiographic (ECG) assessment preoperatively. |
| Proportion of prospective surgical patients that have their full blood count, coagulation profile and renal function checked preoperatively. |

**Intraoperative Indicators**

| Structure Indicators |
| --- |
| Properly designed transfer trolleys meet the requirements of the following list: oxygen cylinders, masks, tubing, infusion poles, equipment to secure and support airway and assist ventilation, provision of clamps for drainage tubes, protective sides, head down tilt possible are available. |
| Equipment to administer oxygen to all patients undergoing procedures under sedation by anaesthesiologists is available. |
| Specialised equipment for the management of difficult airways is available where anaesthesia is given. |
| There is a well-defined internal policy for sedation that includes the training required by the sedation provider, all subspecialty areas and facility specifications and this policy is diffused among professionals. |
| There is a well-defined internal policy that ensures emergency drugs are available where anaesthesia is given and adequately stored defined and this policy is diffused among professionals. |
| There is a well-defined internal policy for the management of complications of anesthesiologist procedures and this policy is diffused among professionals. |
| Devices for maintaining or raising the patient's temperature are available, including control of theatre temperature. ^a^ |
| Defibrillators with cardiac pacing mode are available. |
| Equipment for fluid and blood warming and rapid transfusion is available. |
| Blood storage facilities are in close proximity to emergency theatres and contain 0 rhesus negative blood. |
| There is a well-defined internal protocol for major haemorrhage defined, including clinical laboratory and logistic responses, that is diffused among professionals. |
| Process Indicators |
| An appropriate antibiotic is given as per local guidelines. |
| Equipment used to provide anaesthesia, including monitoring equipment, complies with existing local recommendations. |
| Surgical procedures with predicted mortality >10% are conducted under the direct supervision of a consultant surgeon and anaesthesiologist. |
| The *WHO Surgical Safety Checklist* checklist is applied. |
| Intraoperative blood loss is measured and recorded. ^a^ |
| Surgical pathology specimens are labelled according to recommendations, including: labelled, filled containers, correct laterality, correct tissue type, patient name, and correct patient name. |
| The turnover time between cases is measured. |
| The operating time is recorded. ^a^ |
| Outcome Indicators |
| Failed attempt of endotracheal intubation. |
| Wrong site surgery. ^a^ |
| Intraoperative blood transfusion. |
| Unanticipated transfusion of any blood products. |
| Length of surgery. |

**Postoperative Indicators**

| Structure Indicators |
| --- |
| The Post-Anesthesia Care Unit equipment includes:  - At bedside: pulse oximetry, ECG and Noninvasive Blood Pressure Monitoring  - Immediately available: capnograph, 12 lead ECG, nerve stimulator, thermometer. |
| There is an internal system for ordering, storing, recording and auditing controlled drugs (e.g. morphine, fentanyl) in all postoperative areas where they are used. |
| There is an internal procedure defined for removing endotracheal tubes and supraglottic airways and that is diffused among professionals. |
| There is an internal policy for a member of the anaesthetic/clinical team to visit patients within 24 hours following the surgery (ASA grade 3,4,5: epidural on ward, invasive monitoring in-situ or as requested by health care worker) defined. |
| Internal criteria for discharge from recovery ward are defined and diffused among professionals. |
| Process Indicators |
| The postoperative morphine consumption at 6, 24 and 48 hours is recorded. |
| The analgesic supplementation by any route at 24 hours is recorded. |
| Post-anaesthesia medical records are compliant with local recommendations. The following are recorded: Information about patient evaluation on admission and discharge from Post-Anesthesia Care Unit or admission to the Intensive Care Unit, a time-based record of vital signs and level of consciousness, time-based record of drugs administered, dosage and route of administration, type and counts of intravenous fluids administered, including blood and blood products, and post-anaesthesia visits. |
| The Post-Anesthesia Care Unit's length of stay is recorded. |
| Early warning systems are used at ward care. |
| The discharge destination is recorded. |
| Medical records are compliant with local recommendations. Information about discharge needs assessment and venous thromboembolism prophylaxis is recorded. |
| Recovery area complies with local standards. |
| Outcome Indicators |
| Mortality. |
| Fever. |
| Postoperative sepsis. |
| Septic Shock. |
| Postoperative pneumonia. |
| Antibiotics use. |
| Unplanned return to operating theatre within hospital stay or intervention in the same area or for a reason related to the original intervention. |
| Readmission to the Intensive Care Unit or Intermediate Medical Care Unit. ^a^ |
| Unplanned readmission to hospital. |
| Length of stay. |
| Length of stay in Post-Anaesthesia Recovery Area. |
| Length of stay in the Intensive Care Unit. ^a^ |

#

**Mixed Perioperative Indicators**

| Structure Indicators |
| --- |
| Alternative language leaflets or videos and interpreters appropriate to the needs of the local population are available to patients and caregivers. |
| There is an internal policy for senior clinicians to discuss the defined limits of care and resuscitation that is diffused among professionals. |
| There is a defined internal policy for planned maintenance and replacement programme for anaesthetic equipment defined, including naming a consultant to oversee the provision of anaesthetic equipment that is diffused among professionals. |
| There is an internal policy for anaesthetic emergencies defined and diffused among professionals. |
| There is an internal policy for managing morbidly obese patients defined and diffused among professionals. |
| There is an internal policy for remote site anaesthesia defined and diffused among professionals. |
| There is an internal policy for critical care referral defined and diffused among professionals. |
| There is an internal policy for resuscitation defined and diffused among professionals. |
| There is an internal policy for end of life care defined and diffused among professionals. |
| There is an internal policy for staff training for both technical and non-technical skills defined, including in resuscitation. |
| There is an internal policy for the handover of care of the patient from one team to the other throughout the perioperative pathway defined. ^a^ |
| There is an internal policy for the management and reporting of adverse events and near miss events relating to the perisurgical period. |
| Facilities for rest for on-call/on-duty staff are available. |
| The number of existing theatres (excluding radiology suites, dedicated obstetric, minor operations but including day theatre) is considered adequate according to national recommendations. |
| There is an internal policy for receiving feedback from patients and caregivers, including complaints, in place. |
| Process Indicators |
| Whether pain is measured within the postoperative period, including pre-discharge and after discharge. |
| The length of stay is measured. |
| Outcome Indicators |
| Absence of falls following surgery. |
| Perioperative hypothermia. |
| Perioperative hypoglycemic events. |
| Transfused patients. |
| Venous thromboembolism prophylaxis. |
| Anticoagulation therapy. ^a^ |

^a^ Indicator included in the FLI after the first round of the eDelphi
