# Supplementary material for: Moving towards a core measures set for patient safety in perioperative care: An e-Delphi consensus study
Source: PLoS One. 2024 Oct 23;19(10):e0311896. doi: 10.1371/journal.pone.0311896 (PMC11498713; doi:10.1371/journal.pone.0311896)
Supplement: S1 Table — (DOCX) [file pone.0311896.s002.docx]

### **S1 Table.** Detailed information on the characteristics of the experts.

|  | **n** | **%** |
| --- | --- | --- |
| **Gender** |  |  |
| Female | 22 | 39,3% |
| Male | 31 | 55,4% |
| Non-binary | 0 | 0,0% |
| Prefer not to answer | 3 | 5,4% |
| Total | 56 | 100,0% |
|  |  |  |
| **Age group** |  |  |
| 18-34 | 2 | 3,6% |
| 35-54 | 22 | 39,3% |
| 55-74 | 29 | 51,8% |
| 75+ | 1 | 1,8% |
| Prefer not to answer | 2 | 3,6% |
| Total | 56 | 100,0% |
|  |  |  |
| **Highest Level of Education completed** |  |  |
| Postgraduate education | 50 | 89,3% |
| Tertiary (higher) education | 4 | 7,1% |
| Secondary school | 2 | 3,6% |
| Primary school | 0 | 0,0% |
| None / Incomplete primary school | 0 | 0,0% |
| Prefer not to answer | 0 | 0,0% |
| Total | 56 | 100,0% |
|  |  |  |
| **Role** |  |  |
| **Healthcare professional** | 43 | 76,8% |
| **Profession** |  |  |
| Medical Doctor | 33 | 76,7% |
| Nurse | 5 | 11,6% |
| Quality expert | 2 | 4,7% |
| Hospital manager | 1 | 2,3% |
| Physical therapist | 1 | 2,3% |
| Other | 1 | 2,3% |
| Prefer not to answer | 0 | 0,0% |
| Total | 43 | 100,0% |
| **Area of expertise** |  |  |
| Anaesthesiology | 20 | 46,5% |
| Surgery | 9 | 20,9% |
| Primary Care | 3 | 7,0% |
| Public Health | 3 | 7,0% |
| Haematology | 2 | 4,7% |
| Rehabilitation | 1 | 2,3% |
| Other | 5 | 11,6% |
| Geriatrics | 0 | 0,0% |
| Prefer not to answer | 0 | 0,0% |
| Total | 43 | 100,0% |
| **Patients and/or patient representative** | 6 | 10,7% |
| **Submitted to surgery ≤ 5 years ago** |  |  |
| Yes | 2 | 33,3% |
| No | 1 | 16,7% |
| No, but a direct family member was | 3 | 50,0% |
| Prefer not to answer | 0 | 0,0% |
| Total | 6 | 100,0% |
| **Governmental agency representative** | 3 | 5,4% |
| **Methodologist/Researcher** | 2 | 3,6% |
| **Policymaker** | 1 | 1,8% |
| **Private sector representative** | 1 | 1,8% |
| **Regulatory agencies representative** | 0 | 0,0% |
| **Guideline developer** | 0 | 0,0% |
| **Other** | 0 | 0,0% |
| **Prefer not to answer** | 0 | 0,0% |
| Total | 56 | 100,0% |
|  |  |  |
| **Any Conflict of Interest declared** |  |  |
| Yes | 4 | 7,1% |
| No | 52 | 92,9% |
| Total | 56 | 100,0% |
